# The short-term association of temperature and rainfall with mortality in Vadu Health and Demographic Surveillance System: a population level time series analysis

**DOI:** 10.3402/gha.v5i0.19118

**Published:** 2012-11-23

**Authors:** Vijendra Ingole, Sanjay Juvekar, Veena Muralidharan, Somnath Sambhudas, Joacim Rocklöv

**Affiliations:** 1 Vadu HDSS, KEM Hospital Research Centre, Pune, India; 2 Department of Public Health and Clinical Medicine, Epidemiology and Global Health, Umea University, Umea, Sweden; 3 Indepth Network, Accra, Ghana

**Keywords:** temperature, rainfall, precipitation, climate extreme, extreme weather, HDSS, time series, climate, climate change, weather, precipitation, death, mortality, India

## Abstract

**Background:**

Research in mainly developed countries has shown that some changes in weather are associated with increased mortality. However, due to the lack of accessible data, few studies have examined such effects of weather on mortality, particularly in rural regions in developing countries.

**Objective:**

In this study, we aimed to investigate the relationship between temperature and rainfall with daily mortality in rural India.

**Design:**

Daily mortality data were obtained from the Health and Demographic Surveillance System (HDSS) in Vadu, India. Daily mean temperature and rainfall data were obtained from a regional meteorological center, India Meteorological Department (IMD), Pune. A Poisson regression model was established over the study period (January 2003–May 2010) to assess the short-term relationship between weather variables and total mortality, adjusting for time trends and stratifying by both age and sex.

**Result:**

Mortality was found to be significantly associated with daily ambient temperatures and rainfall, after controlling for seasonality and long-term time trends. Children aged 5 years or below appear particularly susceptible to the effects of warm and cold temperatures and heavy rainfall. The population aged 20–59 years appeared to face increased mortality on hot days. Most age groups were found to have increased mortality rates 7–13 days after rainfall events. This association was particularly evident in women.

**Conclusion:**

We found the level of mortality in Vadu HDSS in rural India to be highly affected by both high and low temperatures and rainfall events, with time lags of up to 2 weeks. These results suggest that weather-related mortality may be a public health problem in rural India today. Furthermore, as changes in local climate occur, adaptation measures should be considered to mitigate the potentially negative impacts on public health in these rural communities.

Climate change is often described as the greatest challenge faced by humanity, with a potentially severe adverse impact on the environment and global population (1). While specific, local outcomes of climate change are uncertain, recent assessments forecast alteration in the frequency, intensity, spatial extent, and duration of weather and climate extremes. This includes climate and hydrometeorological events such as heat waves, heavy precipitation events, drought, and tropical cyclones (2). Some changes in weather will most likely lead to increased stress on human health and environmental systems, both directly and indirectly (2). Weather is commonly identified and studied in terms of sunlight, cloudiness, humidity, precipitation, temperature, and wind, and the local weather depends on the local climate regimen (1). The direct effect of weather, such as extreme temperatures, can disrupt the homeostasis of the body, including the regulation of body heat. Within certain limits, thermal comfort can be maintained by appropriate thermoregulatory responses so that physical and mental activities can be pursued without any damage to health (3). Changes in climate can also lead to proliferation of increasing numbers of vectors known to transmit infectious diseases, or enhance the replication rate of virus and bacteria affecting the transmission of food and waterborne diseases. The prevalence and range of a particular microbe, disease vector, or animal reservoir is dependent on specific ranges of temperature, precipitation, and humidity (4, 5). The geographical and temporal distributions as well as the incidence of many vector-borne diseases (i.e. malaria and dengue), particularly those spending part of their lifecycle outside the human body, are sensitive to temperature and rainfall. Pathogens that are carried by insects are exposed to ambient weather. Vector-borne diseases typically exhibit seasonal patterns in which the role of temperature and rainfall is well reported (17). In India, diarrheal diseases typically peak during the rainy season. Floods and droughts increase the risk of diarrheal diseases, and the major causes of diarrhea associated with heavy rainfall and contaminated water supplies include cholera, cryptosporidium, *Escherichia coli* infection, giardia, shigella, typhoid, and viruses such as hepatitis A (17). Furthermore, cholera outbreaks in coastal areas of Bangladesh have been linked with sea-surface temperature and abundance of plankton, which are thought to be an environmental reservoir for the cholera pathogen (6, 7).

The effects of low temperatures should be stressed as they can result in additional adverse health consequences, including deaths from cardiovascular stress, respiratory disease, impaired mental abilities, and loss of motivation as found in cases of hypothermia (3).

Many studies have been carried out in developed countries, which reported evidence of increased mortality in association with extreme ambient temperatures (9) However, few studies have examined the temperature–mortality relationship in rural areas of developing countries (10, 11). In India, where the heat wave of 1998 was estimated to have caused 1,658 excess deaths, there are grounds for changes in climate to serve as a significant public health concern (12). Studies have reported that excessive rain events that cause flooding can also play an important role in aggravating the public health problems, that is, the spread of water-related communicable diseases, such as diarrhea (13). In India, it was reported that increasing rates of diarrhea disease, also including cholera, are related to poor sanitation facilities and extreme rainfall events (9). Diarrhea is a major cause of mortality among children under 5 years of age in India and is considered to be a significant public health problem (14). A study in Bangladesh, investigating the effects of floods on health, found that the size of the family and low economic status were associated with higher diarrhea incidents (15). During 2000 and 2001 in Mumbai, outbreaks of leptospirosis were reported in children living in informal settlements after floods and the prevalence of leptospirosis increased eight-fold following the major flood event in July 2005 (18). Studies on hospital-based observation found that the risk of disease was associated with children either playing in the floodwater or wading through it while going to school and, in some cases, where floodwater was inside the house (19).

The aforementioned studies suggest that a significant amount of work has already been done. However, there are very few studies that have assessed and quantified the association between population level mortality and exposure to temperature and rainfall in rural populations of developing countries.

## Objectives

The aim of this study was to estimate the short-term immediate and delayed association of temperature and rainfall on daily mortality in different strata of age and sex in Vadu HDSS and to quantify relative risk per lag strata by groups of age and sex.

## Materials and methods

### Study area and population

Vadu HDSS is a member of the International Network for the Demographic Evaluation of Populations and Their Health (INDEPTH) in developing countries, a global network of centers that conduct longitudinal health and demographic evaluation of populations in low- and middle-income countries. Vadu HDSS covers 22 villages from two administrative blocks in the Pune District of India. Its geographical extent is 18°30′ to 18°47′ N Latitude and 73°58′ to 74°12′ E Longitude, covering a 232 km^2^ geographical unit, with an average altitude of 560 m. Winter lasts from November to February and is followed by a summer that lasts up to early June. The monsoon in the Vadu HDSS area starts from early June and continues until the beginning of October. The latter part of October is the post-monsoon season. November to February is winter. After February, the temperature rises rapidly until April or May corresponding to the hottest months of the year, on average. While days are generally hotter during April with a mean daily maximum of 40–42°C, nights are warmer during May and June with a mean daily minimum of 23–24°C. Toward the end of the monsoon in October, there is a slight increase in the day temperature, but the nights become progressively cooler. December is the coldest month with mean daily minimum temperature of about 12–13°C (India Meteorological Department [IMD] Pune, 2010). Among the total population of approximately 100,000, about 46% are workers employed in the manufacturing sector, service industry, or non-household industry, whereas 20% are cultivators. Of the total population, 23% work at home, and 11% are students or children not in school. The female to male ratio in Vadu HDSS for the total population was 770: 1,000 over the period of January 2003 to May 2010.

### HDSS data collection

Since 2003, the HDSS data have been collected biannually from January to June and July to December. Field research assistants (FRA) visit every household in all villages to record demographic events, including births, deaths, in-migrations, out-migrations, and pregnancies within the Vadu HDSS area. Each event is recorded for all families residing within the area using questionnaires administered by the FRAs who are also local residents. Hence, for the current analysis, information on sex and date of death, and reported cause of death was retrieved from the HDSS data over the study period. The deaths reported during the study period were 1,662. We stratified the number of daily deaths in groups by age, 0–4, 5–19, 20–59, 60+, and by males and females over the study period. The total number of deaths in all groups as well as the maximum and minimum per day over the study period are presented in Table 1.

**Table 1:** Mortality frequencies stratified by groups of age and sex in Vadu HDSS, 2003–2010

Age-group	Daily maximum	Daily minimum	Total mortality
0–4 years	2	0	46
5–19 years	2	0	62
20–59 years	4	0	627
>60 years	13	0	927
Men	8	0	954
Women	10	0	708
Total	18	0	1,662

### Meteorological data

Daily weather data were obtained from IMD Pune for a period of 8 years (January 2003–May 2010). We obtained recordings of the mean daily temperature and cumulative daily precipitation (Table 2).

**Table 2:** Descriptive statistics of daily meteorologic measurement in Vadu HDSS, 2003–2010

Meteorological variables	Mean (°C)	Maximum (°C)	Minimum (°C)	Std. dev.
Daily maximum temperature	32.2	42.4	21.1	3.9
Daily minimum temperature	18.3	28.0	4.7	4.7
Daily mean temperature	25.2	34.9	15.5	3.2
Rainfall (mm)	1.3	95.0	0.0	6.5

### Statistical analysis

We used daily mean temperature and daily cumulative rainfall as explanatory variables of daily mortality. We examined the relationship between daily mortality and the weather variables using time series Poisson regression models allowing for over-dispersion. We used a smooth cubic spline function to adjust for season and time trends allowing six degrees of freedom (df) per year of data over the study period. The short-term weather mortality relationship was estimated using smooth penalized cubic spline functions to avoid too complex fits. Exposure–response functions were also estimated linearly for temperature and rainfall and indicated little deviation from linearity. Potential delayed effects from the weather variables on mortality were assessed via lag strata and averaged the temperature versus rainfall over the periods of lag 0–1, lag 2–6, and lag 7–13 days The purpose was to avoid collinearity introduced by having several highly correlated explanatory variables in the regression model simultaneously.

The models used for this analysis could be expressed as:

$$\begin{array}{ll} {\text{Deaths}}_{\text{t}}{\sim \text{Poisson (mean}}_{\text{t}}\text{)} & \\ {\text{log (mean}}_{\text{t}}\text{)} & \text{= intercept + s(temperature lag 0}–\text{1, df = 4)}\\ & \text{+ s(temperature lag 2}–\text{6, df = 4)}\\ & \text{+ s(temperature lag 7}–\text{13, df = 4)}\\ & \text{+ s(rain lag 0}–\text{1, df = 4)}\\ & \text{+ s(rain lag 2}–\text{6, df = 4)}\\ & \text{+ s(rain lag 7}–\text{13, df = 4)}\\ & \text{+ s(time, df = 6 per year of data).} \end{array}$$

And,

$$\begin{array}{ll} {\text{log (mean}}_{\text{t}}\text{)} & \text{= intercept + temperature lag 0}–\text{1}\\ & \text{+ temperature lag 2}–\text{6}\\ & \text{+ temperature lag 7}–\text{13}\\ & \text{+ rain lag 0}–\text{1}\\ & \text{+ rain lag 2}–\text{6}\\ & \text{+ rain lag 7}–\text{13}\\ & \text{+ s(time, df = 6 per year of data).} \end{array}$$

Where ‘s’ denotes a cubic spline function with ‘df’ number of degrees of freedom (df), and ‘t’ denotes the time of observation. The autocorrelation function was estimated and assessed to examine potential residual confounding patterns. Relative risks (RR) corresponding to a 1°C increase in temperature and a 1 mm increase in rainfall are presented with 95% confidence limits for the linear estimates.

## Results

**Fig. 1:**
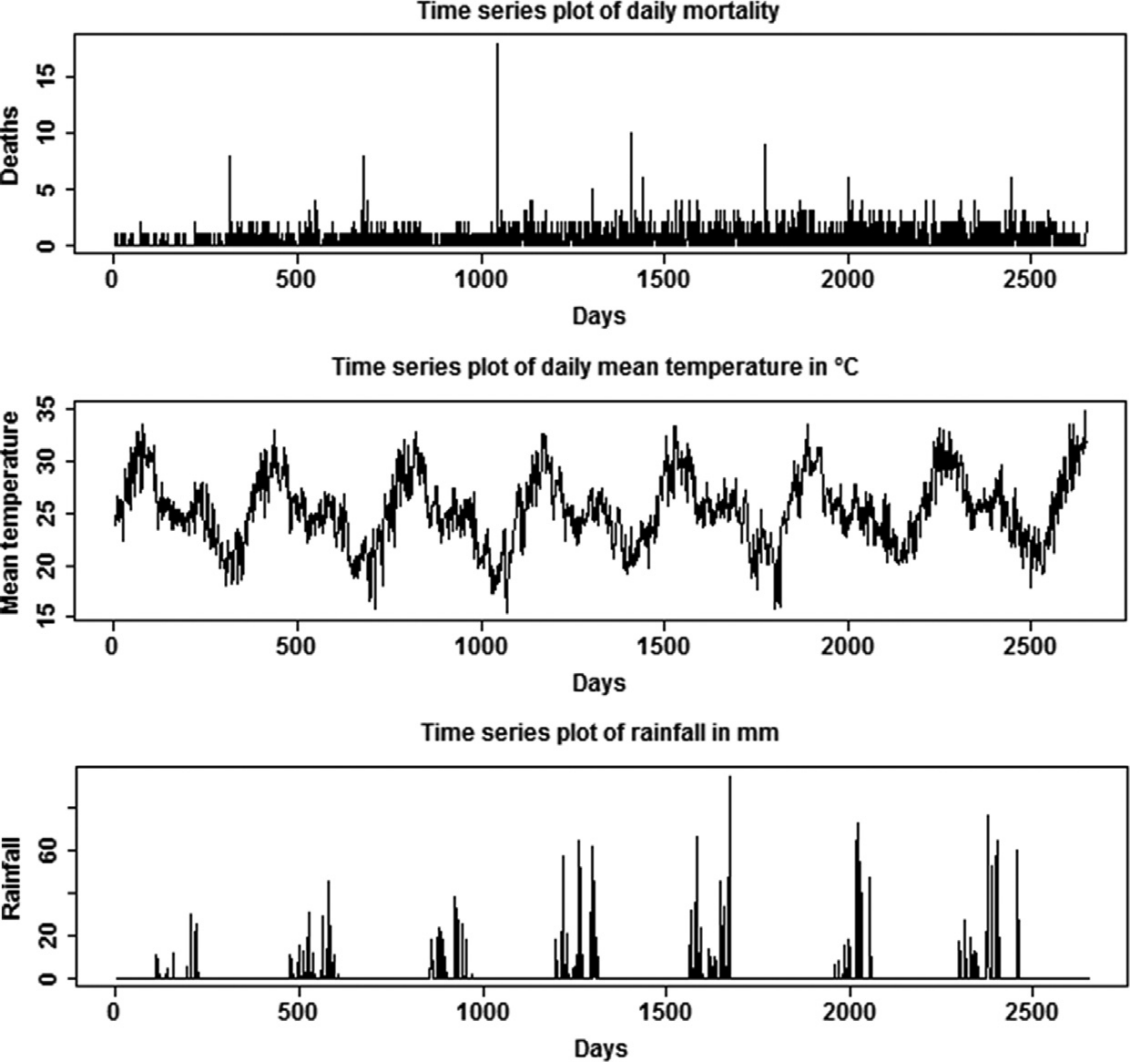
Time series plot of daily mortality, daily mean temperature (°C), and rainfall (mm) in Vadu HDSS, 2003–2010.

The time series plots for daily mortality, daily mean temperature, and cumulative rainfall are presented in Fig. 1. Temperature is strongly seasonal with peaks in March, April, and May, and rainfall shows an increasing trend and peaks during the months of June, July, and August. The highest peak indicated by the plot is for August 2008 where rainfall was >90 mm in a single day.

**Fig. 2:**
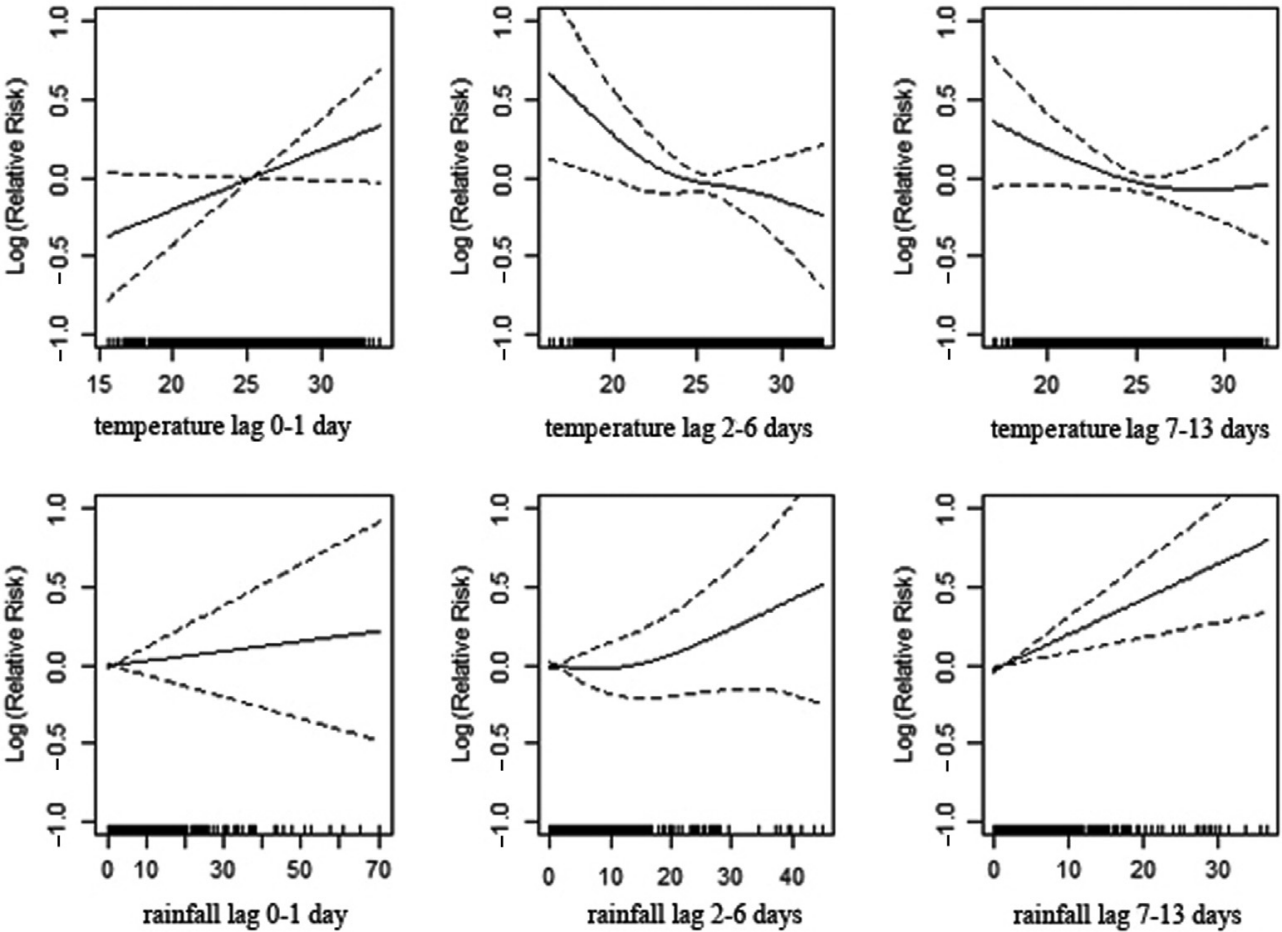
Association of mortality with daily temperature and rainfall in Vadu HDSS, 2003–2010.

Figure 2 shows the association between temperature and rainfall with daily mortality in total population over all time lagged strata studied. These associations indicate that elevated temperature drives effects in lag 0–1, whereas depressed temperatures drive the trend in lags 2–6. A strong positive correlation is evident between the onset of rainfall and subsequent mortality for the period of 7–13 days.

**Fig. 3:**
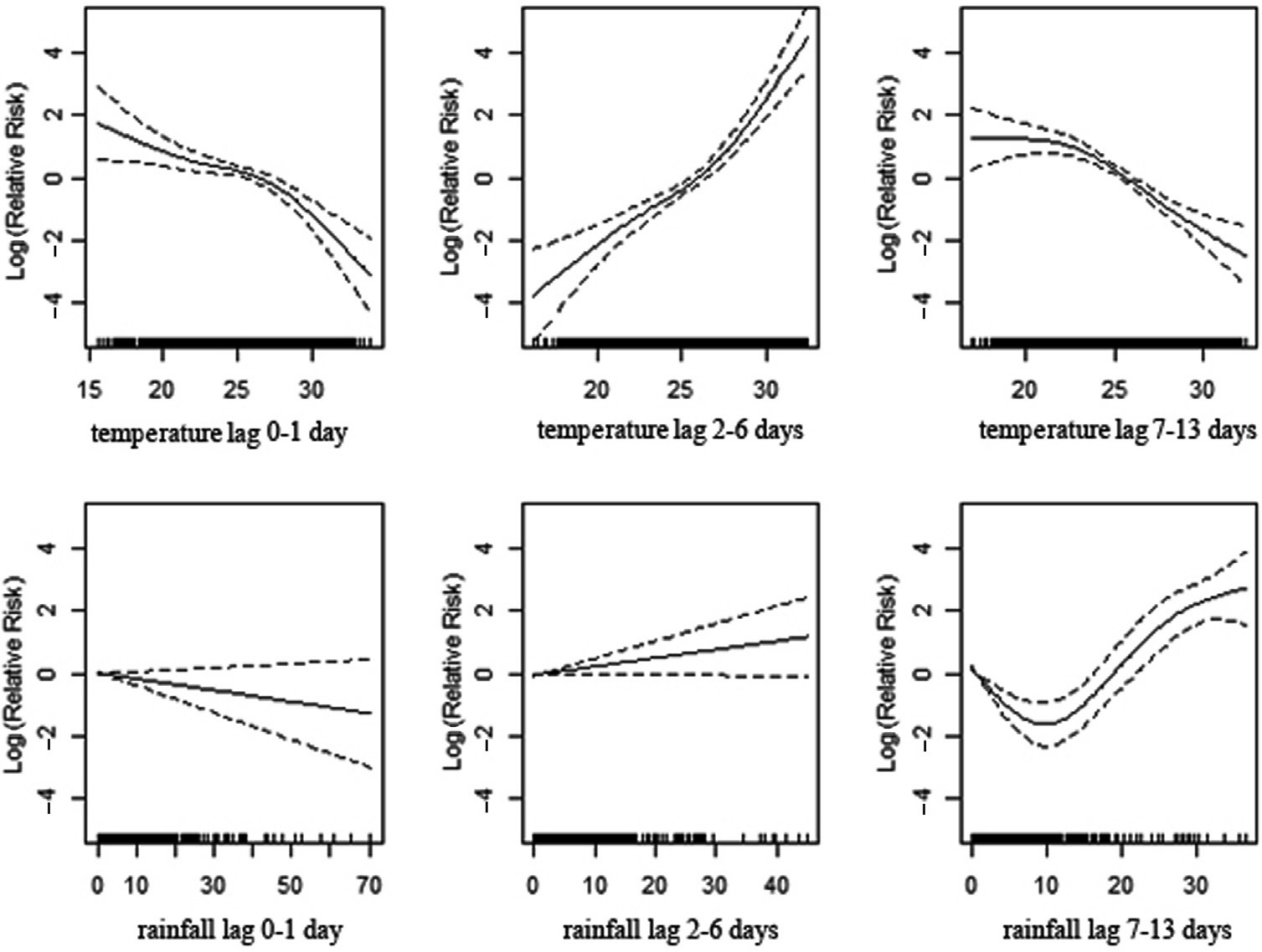
Association of mortality with daily temperature and rainfall in the age strata of 0–4 years in Vadu HDSS, 2003–2010.

**Fig. 4:**
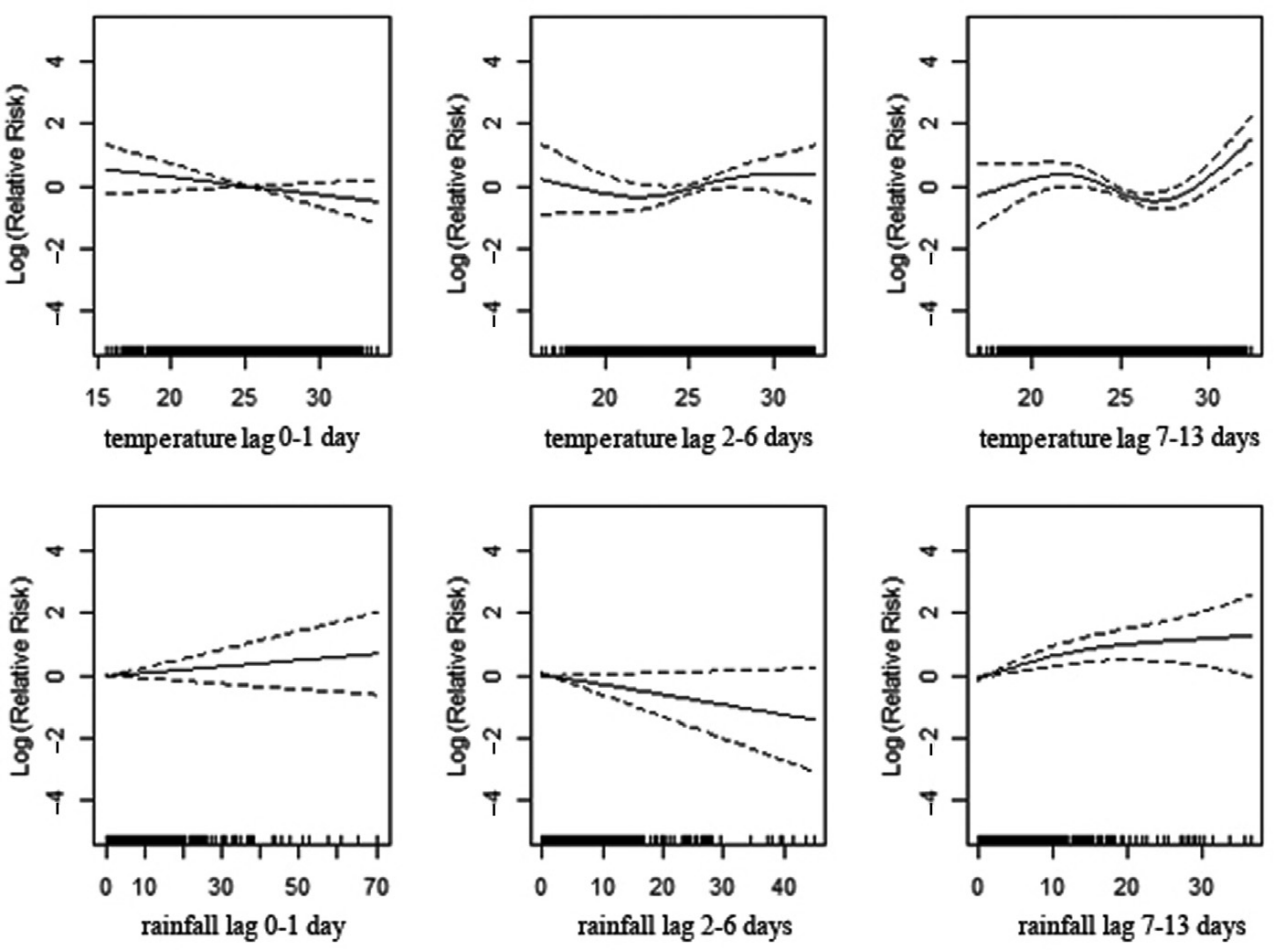
Association of mortality with daily temperature and rainfall in the age strata of 5–19 years in Vadu HDSS, 2003–2010.

**Fig. 5:**
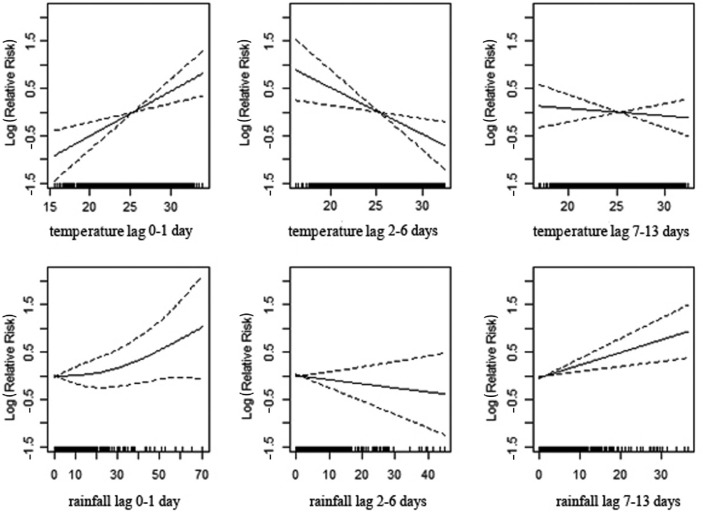
Association of mortality with daily temperature and rainfall in the age strata of 20–59 years in Vadu HDSS, 2003–2010.

**Fig. 6:**
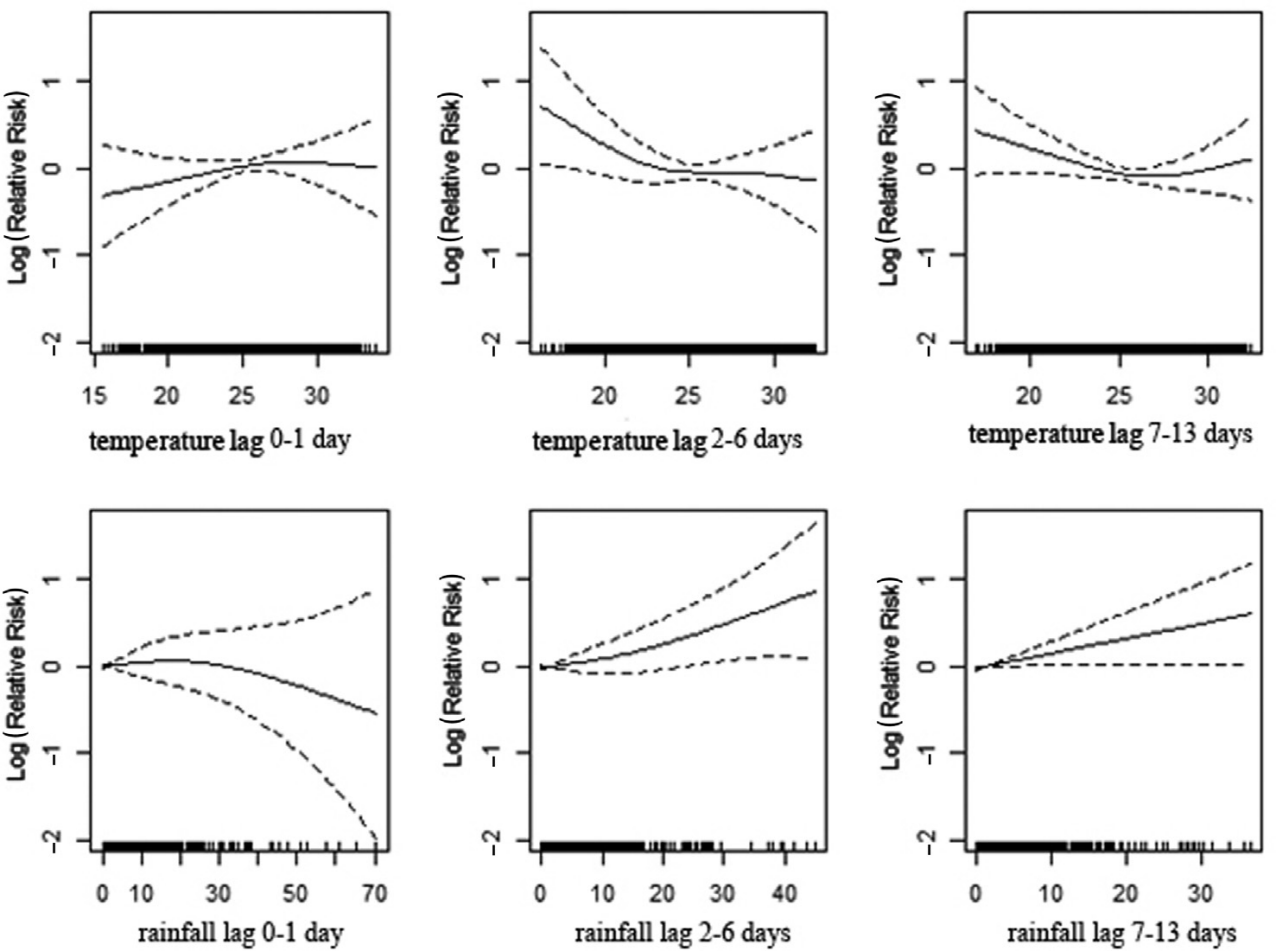
Association of mortality with daily temperature and rainfall at the age of 60 years in Vadu HDSS, 2003–2010.

**Fig. 7:**
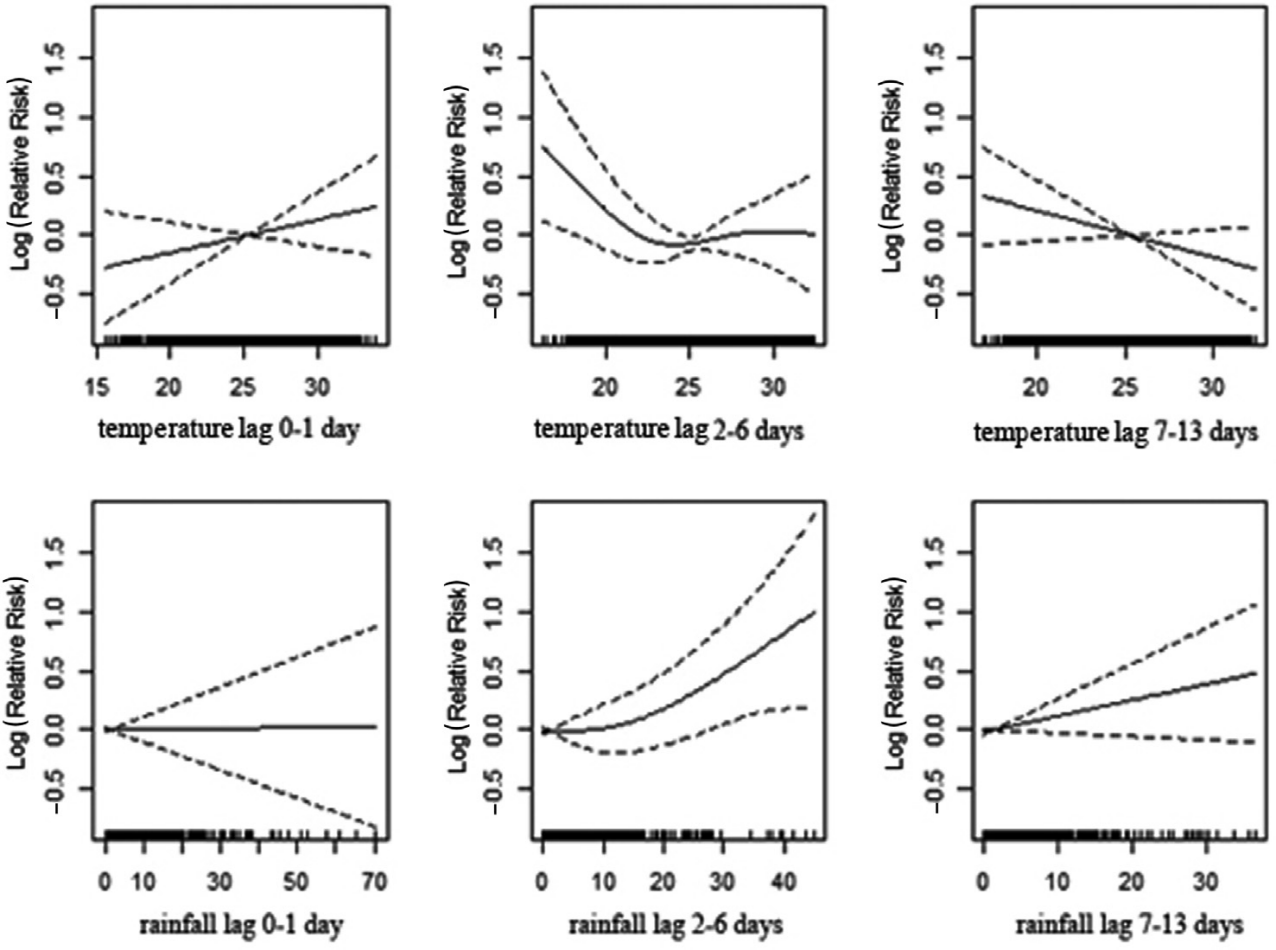
Association of mortality among men and daily temperature and rainfall in Vadu HDSS, 2003–2010.

**Fig. 8:**
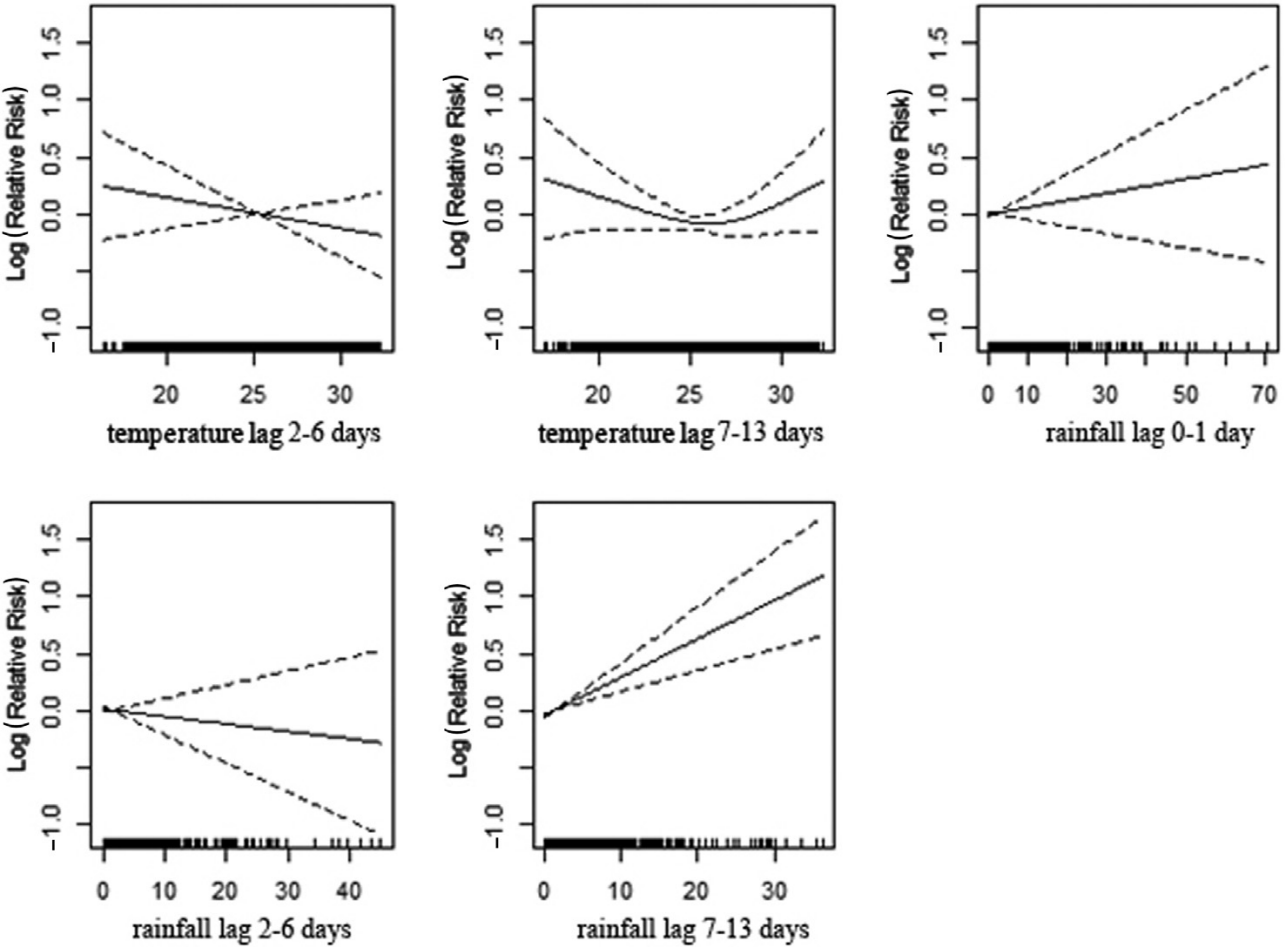
Association of mortality among women and daily temperature and rainfall in Vadu HDSS, 2003–2010.

To better understand the relationship among daily mortality, mean temperature, and cumulative rainfall, we created regression models for mortality, stratifying by age and sex. (Table 3; Fig. 3), shows a significant association between mortality and temperature for the age group of 0–4 years, apparent with lower temperature in lag 0–1 and 7–13 and with high temperature in lag 2–6. High levels of rainfall significantly increase deaths among children with a 1–2 week delay. In the age group 5–19 (Table 3; Fig. 4), the lag 7–13 temperatures show positive associations with mortality during extreme hot conditions. However, the association is weaker compared to the younger age group. Increasing amounts of rainfall in lag 7–13 were strongly associated with increasing mortality while a reduction in deaths was observed during the first week following rainfall (Table 3; Fig. 4). In the age group 20–59 (Table 3; Fig. 5), a positive association is reported with higher temperatures in lag 0–1, whereas the opposite pattern is present in lag 2–6. Rainfall shows a positive association with mortality in lag 0–1 and, in particular, a strong significant association in 1–2 weeks after rainfall. The elderly appear susceptible to increasing rainfall (lag 2–13). However, there are no strong apparent patterns associated with temperature in this age group (Table 3; Fig. 6). Figures 7 and 8 and Table 4 show the corresponding associations in the groups of men and women. The graphs and Table 4 indicate that women may be more susceptible to the mortality effects following rainfall events (lag 7–13) compared to men.

**Table 3:** Relative risks (% per degree increase) and 95% confidence intervals for temperature and rainfall in different lag periods stratified by age (bold indicates significances at 5% level)

**Age 0–4 years**	**RR (%)**	**CI (%)**
Temperature lag 0–1	**−18.8**	**(−24.9, −12.2)**
Temperature lag 2–6	**66.5**	**(50.0, 84.9)**
Temperature lag 7–13	**−29.3**	**(−36.6, −21.1)**
Rainfall lag 0–1	**−3.6**	**(−5.7, −1.5)**
Rainfall lag 2–6	0.5	(−1.9, 3.0)
Rainfall lag 7–13	−0.1	(−2.8, 2.5)

**Age 5–19 years**	**RR (%)**	**CI (%)**

Temperature lag 0–1	−2.3	(−10.0, 6.2)
Temperature lag 2–6	7.1	(−9.7, 19.1)
Temperature lag 7–13	**15.7**	**(5.0, 27.5)**
Rainfall lag 0–1	1.1	(−1.0, 3.3)
Rainfall lag 2–6	**−6**	**(−1.8, −1.0)**
Rainfall lag 7–13	**6**	**(3.0, 9.2)**

**Age 20–59 years**	**RR (%)**	**CI (%)**

Temperature lag 0–1	**9.4**	**(3.6, 15.5)**
Temperature lag 2–6	**−9.5**	**(−15.5, −3.2)**
Temperature lag 7–13	1.8	(−4.1, 8.1)
Rainfall lag 0–1	0.7	(−0.3, 1.9)
Rainfall lag 2–6	−1.1	(−3.0, 7.5)
Rainfall lag 7–13	**3**	**(1.3, 4.0)**

**Age >60 years**	**RR (%)**	**CI**

Temperature lag 0–1	2.9	(−2.1, 8.0)
Temperature lag 2–6	−3.3	(−1.8, 2.8)
Temperature lag 7–13	2	(−3.4, 7.8)
Rainfall lag 0–1	−0.5	(−1.7, 0.7)
Rainfall lag 2–6	0.8	(−0.6, 2.2)
Rainfall lag 7–13	0.4	(−1.2, 2.0)

**Table 4:** Relative risks (% per degree increase) and 95% confidence intervals for temperature and rainfall in different lag periods stratified by sex (bold indicates significances at 5% level)

**Men**	**RR (%)**	**CI (%)**
Temperature lag 0–1	3.1	(−1.7, 8.1)
Temperature lag 2–6	−2.3	(−8.2, 3.8)
Temperature lag 7–13	2	(−3.4, 7.6)
Rainfall lag 0–1	−0.2	(−1.5, 0.9)
Rainfall lag 2–6	0.8	(−0.6, 2.2)
Rainfall lag 7–13	2.2	(−1.4, 1.9)

**Women**	**RR (%)**	**CI (%)**

Temperature lag 2–6	−6.5	(−12.6, 0.1)
Temperature lag 7–13	1.5	(−4.3, 7.7)
Rainfall lag 0–1	0.3	(−0.9, 1.5)
Rainfall lag 7–13	**2.3**	**(0.7, 3.9)**

## Discussion and conclusion

The present study was designed to determine the associations of mortality with temperature and rainfall. The results of this study primarily indicate that strong associations with temperature and rainfall exist for all-cause mortality over all age groups. The effects could be seen for both high and low temperatures, and up to 2 weeks following rainfall. In particular, the associations were strongest among children, women, and the elderly. In this respect, it appears that the population of Vadu HDSS in rural India is particularly vulnerable to changing weather conditions. It is important to increase resilience to weather, climate change, and climate extreme events in these regions to achieve the millennium development goals (MDGs) and to mitigate harmful effects (8).

It is well known that extreme temperature and rainfall are potent risk factors for certain diseases, including heat stroke, dengue, malaria, and cholera (20). There is further evidence that disease incidences increase during heavy rainfall, as flood water could mix with drinking water, resulting in an increase in mortality (20).

Future studies should elaborate more on the environmental risk factors on a daily basis and their relationship to mortality so as to understand and mitigate potential negative health effects. There is also a need for more studies within this population to describe the roles of socioeconomic and physiologic factors in relation to weather and mortality. To refine the studies, there is also a need to improve environmental monitoring and surveillance systems in developing countries. Research initiatives could focus on long-term data collection on climate-related mortality with the aim of understanding current weather-related associations with mortality and to predict future scenarios. Health outcomes of interest, for which such data should be collected, include total morbidity and mortality and non-communicable diseases, such as cardiovascular, respiratory, circulatory diseases, and asthma, as well as infectious diseases, such as cholera, malaria, tuberculosis, typhoid, hepatitis, and other vector-borne and waterborne diseases. So far in India, health impacts of climate change have not been studied much in detail. However, it is a known fact that the current burden of climate is related to diseases (16). In summary, this study clearly indicates the value of the HDSS for such a purpose and the potential to further refine and identify susceptible groups and hazardous climate-related events to increase resilience of the rural communities to these impacts.
